# Unraveling candidate genomic regions responsible for delayed leaf senescence in rice

**DOI:** 10.1371/journal.pone.0240591

**Published:** 2020-10-15

**Authors:** Uma Maheshwar Singh, Pallavi Sinha, Shilpi Dixit, Ragavendran Abbai, Challa Venkateshwarlu, Annapurna Chitikineni, Vikas Kumar Singh, Rajeev K. Varshney, Arvind Kumar

**Affiliations:** 1 International Rice Research Institute (IRRI), South Asia Hub, ICRISAT, Hyderabad, India; 2 South Asia Regional Centre (ISARC), International Rice Research Institute, Varanasi, India; 3 Centre of Excellence in Genomics and Systems Biology, International Crops Research Institute for the Semi-Arid Tropics (ICRISAT), Hyderabad, India; Faculty of Agriculture (FoA), Sher-e-Kashmir University of Agricultural Sciences and Technology of Kashmir (SKUAST-K), Wadura Campus, INDIA

## Abstract

Photosynthates generated after heading contributes to 60% - 80% of grain yield in rice. Delay in leaf senescence can contribute to a long grain-filling period and thereby increased yield. The objective of this study was to identify genomic region(s) responsible for delayed leaf senescence (DLS) and validate the role of underlying candidate genes in controlling target traits. 302 BC_2_F_4_ backcross-derived lines (BILs) developed from a cross between Swarna and Moroberekan were phenotyped for two seasons (DS2016 and WS2017) for chlorophyll content and yield parameters. KASPar-SNP assays based genotyping data with 193 SNPs of mapping population was used to identify the targeted genomic region(s). Significant positive correlation was observed between the two most important determinants of DLS traits *viz*., RDCF (reduced decline degree of chlorophyll content of flag leaf) and RDCS (reduced decline degree of chlorophyll content of second leaf) with plant height (PH), grain number per panicle (GPN), panicle length (PL), number of tiller (NT) and grain yield (GY). A total of 41 and 29 QTLs with phenotypic variance (PVE) ranging from 8.2 to 25.1% were detected for six DLS traits during DS2016 and WS2017, respectively. Out of these identified QTLs, 19 were considered as stable QTLs detected across seasons. 17 of the identified stable QTLs were found to be novel. *In-silico* analysis revealed five key genes regulating chlorophyll metabolism. Expression analysis of these genes confirmed their strong association with the senescence pattern in leaf tissue of parents as well as selected phenotypically extreme lines. The identified stable QTLs regulating DLS traits and validation of potential candidate genes provides insight into genetic basis of delayed senescence and is expected to contribute in enhancing grain yield through genomics-assisted breeding (GAB).

## Introduction

Rice is one of the world’s most important food crops, especially in the developing world. Rice production has steadily increased during the green revolution, but in recent years the rate of increase in grain yield has substantially reduced [1]. To increase grain yield in cereal crops, source strength needs to be increased so that sink organs can be more efficiently filled *via* efficient nutrient translocation. Delay in the onset of leaf senescence or slower degradation of chlorophyll content and photosynthetic activity is expected to extend the assimilatory capacity of the canopy thereby contributing to higher grain yield [2]. Photosynthates generated after heading contributes to 60% - 80% of grain yield in rice and are vital for grain production [3].

Leaf senescence is complex and highly regulated process that involve dismantling of chloroplast, decline in photosynthetic activity and degradation of macromolecules such as nucleic acid, protein and lipids [4]. During senescence nutrient are re-mobilized from sensing leaves to other plant parts such as developing seeds and storage organs [5]. Due to its role in the efficient use of nutrients, which is essential for high crop yields, senescence has attracted special interest especially in monocarpic plants. Positive correlation between crop yield and duration of green leaf area in rice has been reported [4–7]. The role of stay green has also been established in enhancing drought tolerance in EMS induced mutant of N22 rice [8]. Therefore, breeding for delayed leaf senescence (stay-green) traits could be of strong interest to crop breeders.

Jiang et al. [9] classified stay-green into two groups: functional stay-green and non-functional stay-green. The functional stay-green trait is beneficial for extending the assimilatory capacity of the canopy and improving crop yield potential; it is classified into type A (a delay in the onset of leaf senescence) and type B (a slower decrease in the rate of photosynthesis) [10]. Functional stay-green genotypes have been drawing more and more attention due to increased photosynthetic capacity in the final stage of plant growth, and consequently higher grain yield [11]. On the other hand, non-functional stay-green genotypes have been reported not to increase photosynthetic activity, although the leaves remain green till the final stage of plant development [11,12].

Genetic dissection of target traits through mapping and transcript analysis is currently a powerful approach for better understanding of complex traits including delayed leaf senescence. It has been demonstrated that stay-green is largely polygene in nature regulated by quantitative traits [3]. Genetic mapping of quantitative trait loci (QTLs) for stay-green has been conducted in many crops including rice [6,9,11–13], sorghum [14–18] maize [19–22].

In rice, germplasm exhibiting stay-green characteristics at maturity have been identified such as Wuyujing2 [9], IRAT109 [12], SNU-SG1 [4], Fedearroz 50 [23] and Akenohoshi [5] and some of these have been used in QTL mapping. However, most of the mapping studies reported a negative correlation of stay green with grain yield in rice except few study like SNU-SG1/Ilpumbyeo [4] and Akenohoshi/ Koshihikari [5].

In the current study, a backcross derived (BC_2_F_4_) mapping population generated from cross of the delayed senescence *indica* rice “Swarna” and early senescent *japonica* rice “Moroberekan” was phenotyped for traits influencing delayed leaf senescence to (1) study the genetic correlation between the delayed leaf senescence-related traits and yield components (2) identify genomic region(s) associated with delayed leaf senescence-related traits (3) identify genes for delayed leaf senescence/chlorophyll catabolism in QTL regions and validate their roles through expression study (4) identification of superior breeding lines with higher yield and delayed leaf senescence.

## Materials and methods

### Plant materials and development of mapping population

The experimental population consists of 302 BC_2_F_4_ derived-backcross inbred lines (BILs) developed using single seed descent (SSD) method from a cross between Swarna as recipient (lowland *indica* rice variety with delayed senescence) and Moroberekan as donor (upland traditional *japonica* rice variety with early senescence) [24]. The population was analyzed in the wet season (WS2016) and dry season (DS2017) under transplanted conditions using an alpha lattice design in two replications at a spacing of 20 × 15 cm and standard agronomic management practices were followed.

### Phenotyping of delayed senescence, yield and related components

Six component traits that influence delayed leaf senescence were analyzed to address delayed leaf senescence traits. The degree of chlorophyll content/SPAD (Soil Plant Analysis Development) Value of flag leaf (FL) and second leaf (SL) was measured at heading were designated as degree of chlorophyll content of flag leaf (1DCF) and degree of chlorophyll content of second leaves (1DCS), respectively. SPAD value of FL and SL at 30 days after heading (DAH) is designated as 30DCF and 30DCS respectively. Relative decline degree of chlorophyll content of FL and SL was calculated by dividing SPAD value at 30 days after heading with SPAD value at heading deducted by one is designated as RDCF and RDCS respectively. To evaluate delayed leaf senescence, we recorded 1DCF, 30DCF, 1DCS and 30DCS of parents (Swarna, Moroberekan) and BC_2_F_4_ derived mapping population. The chlorophyll content of FL and SL of randomly selected three plants were measured (between 12:00 and 15:00) at heading and 30 days post heading, with Minolta Chlorophyll Meter SPAD-502 (Minolta, Japan), an indirect indicator of chlorophyll density during both the seasons. To ensure recorded measurements were from the same tiller and day, tillers were tagged on the day of heading. SPAD reading was performed in three replications by measuring one leaf per plant and at least three parts of the leaf.

In addition, yield and yield-related traits such as DTF, PH, TN, PL, GN, TW and GY (kg h^-1^) were recorded in both WS2016 and DS2017. DTF was recorded at 50% of flowering, PH, NT and PL were measured from randomly selected three plants at maturation stage, whereas GN and TW and GY was recorded post-harvest.

### Genotyping, construction of linkage map and QTL mapping

Mapping population was genotyped using KASPar SNP assays (Dixit et al. 2014). A total of 2,015 SNP markers were screened between the two parents for getting polymorphic markers. Lines with percentages higher than predicted for heterozygous alleles (> 40 percent), Moroberekan alleles (> 40 percent), or incomplete data (> 30 percent) were filtered before analysis to maintain data quality. A total of 193 polymorphic markers spaced uniformly across the genome were used to generate the genotypic data of the population and linkage map was constructed which is reported in previous study [24]. Linkage map consists of 193 SNP marker loci covering all 12 chromosomes and spanning 1,525 cM with an average interval of 7.86 cM between markers (S5–S7 Tables in S2 Data). QTL analysis was done using QGene 4.3.10 with the composite interval mapping (CIM) procedure (Joehanes and Nelson 2008). Standard threshold LOD (logarithm of odds) score of 2.5 was used to suggest the presence of a putative QTL. Abbreviations namely, *q1Dcf*, *q30Dcf* were used to indicate QTLs for degree of chlorophyll content of flag leaf at heading and 30 days after heading; *q1Dcs*, *q30Dcs* were used to indicate QTLs for degree of chlorophyll content of second leaf at heading and 30 days after heading; *qRdcf* and *qRdcs*, relative decline in chlorophyll content of flag leaf, and relative decline in chlorophyll content of second leaf, respectively.

### *In silico* mining of potential candidate genes localized in identified QTLs

Firstly, all the genes localized within the identified stable major effect 10 QTL regions (on 7 chromosomes) were fetched. Sequences of markers flanking QTLs on chromosome 1, 2, 3, 5, 6, 7 and 10 were subjected to batch retrieval from IRRI-galaxy resource (http://galaxy.irri.org/), followed by visualization in MapMan [25] for functional categorization. Then, the potential candidate genes localized within them were shortlisted based on literature mining. The SNPs in the selected putative genes were detected between genome sequence of Swarna (CX94) and Moroberekan (C009) using RiceVarMap v2.0 browser (http://ricevarmap.ncpgr.cn/v2/).

### Expression profiling of identified target genes

Total RNA was isolated using Plant RNA Miniprep kit, XcelGen (XG661-01, Xcelris, India) from homogenized tissue of 30 days old (from heading date) FL and SL of phenotypically extreme lines (ELS- early leaf senescence and DLS- delayed leaf senescence) along with parents. The integrity of RNA samples was assessed on 0.8% agarose/formaldehyde gel electrophoresis. The concentration of each sample was checked on the NanoDrop 8000 Spectrophotometer (Thermo Scientific, USA) and three micrograms of RNA was used for first-strand cDNA synthesis using the SuperScript III RT enzyme (Invitrogen, USA) following the manufacturer’s guidelines. The RNA samples with 260/280 ratio of 1.9–2.0 were used to synthesize cDNA samples from the three biological replicates.

The gene-specific primers were designed using Primer-Blast (NCBI, https://www.ncbi.nlm.nih.gov/tools/primer-blast/) a program developed by NCBI that uses the algorithm Primer3 [26,27] with default parameters for qRT-PCR (S8 Table in S2 Data). The qPCR reactions were performed using SYBR green master-mix in 96 well-plates with three biological replicates and technical replicates using *Osubq5* (Os01g0328400) as the endogenous control. The PCR conditions used were as follows: 2 min at 50°C, 10 min at 95°C, and 40 cycles of 15 s at 95°C, and 1 min at 60°C. The relative transcriptional level in terms of fold-change was calculated using the 2^−ΔΔCT^ method [28] and student's t-test was used to calculate significance.

### Statistical procedures

Descriptive analysis was carried out using IBM SPSS v20. Pearson correlation coefficients were calculated for each trait using R software version 3.0.1. Level of significance of gene expression data was tested by student T-test.

## Results

### Phenotypic characterization of population for delayed senescence trait

The SPAD value (indicator of chlorophyll content) of FL and SL was high at heading and thereafter decreased in parents and in the mapping population lines. This reduction was faster in FL of Moroberekan (30.7% reduction) as compared to Swarna (13.7% reduction), similar trend was observed in the case of SL also *viz*. Moroberekan (30.3% reduction) and Swarna (18.5% reduction). Descriptive statistics of the delayed leaf senescence-related trait for parental lines and the mapping population are shown in Table 1. The phenotypic analysis of BC_2_F_4_ derived lines demonstrated that the population exhibited an almost normal distribution for all the related delayed leaf senescence traits (S1 Fig in S1 Data). All skewness and kurtosis values were less than one except RDCF and RDCS occurring for all measured traits (Table 1).

**Table 1:** Descriptive statistics of the six traits for parents and across BC_2_F_4_ derived lines.

Trait^†^	Parents (SPAD value^‡^)		Population					
	Moroberekan	Swarna	Range	Minimum	Maximum	Mean	Skewness	Kurtosis
1DCF	42.79	36.74	15.57	29.3	44.87	37.17 ±.82	-0.61	0.67
1DCS	44.36	40.14	14	33	47	39.43±.83	-0.38	-0.98
30DCF	29.64	31.71	31	14	45	28.10±.92	-0.61	-0.65
30DCS	30.90	32.71	29	12	41	26.35±.94	-0.22	-1.21
RDCF	0.31	0.14	0.33	0.74	0.07	0.32±.03	0.80	1.56
RDCS	0.30	0.19	0.4	0.75	0.09	0.33±.03	1.38	1.35

^†^Average value of phenotypic data from DS2016 and WS2017;

^‡^SPAD value (Index of relative chlorophyll content). 1DCF- degree of chlorophyll content of flag leaf at heading; 30DCF- degree of chlorophyll content of flag leaf 30 days after heading, RDCF-reduced decline in degree of chlorophyll content of flag leaf; 1DCS- degree of chlorophyll content of second leaf at heading; 30DCS- degree of chlorophyll content of second leaf 30 days after heading; RDCS- reduced decline in degree of chlorophyll content of second leaf in BC_2_F_4_ derived lines from Swarna (S) × Moroberekan (M)

### Relation between delayed leaf senescence and yield-related traits

**Fig 1:**
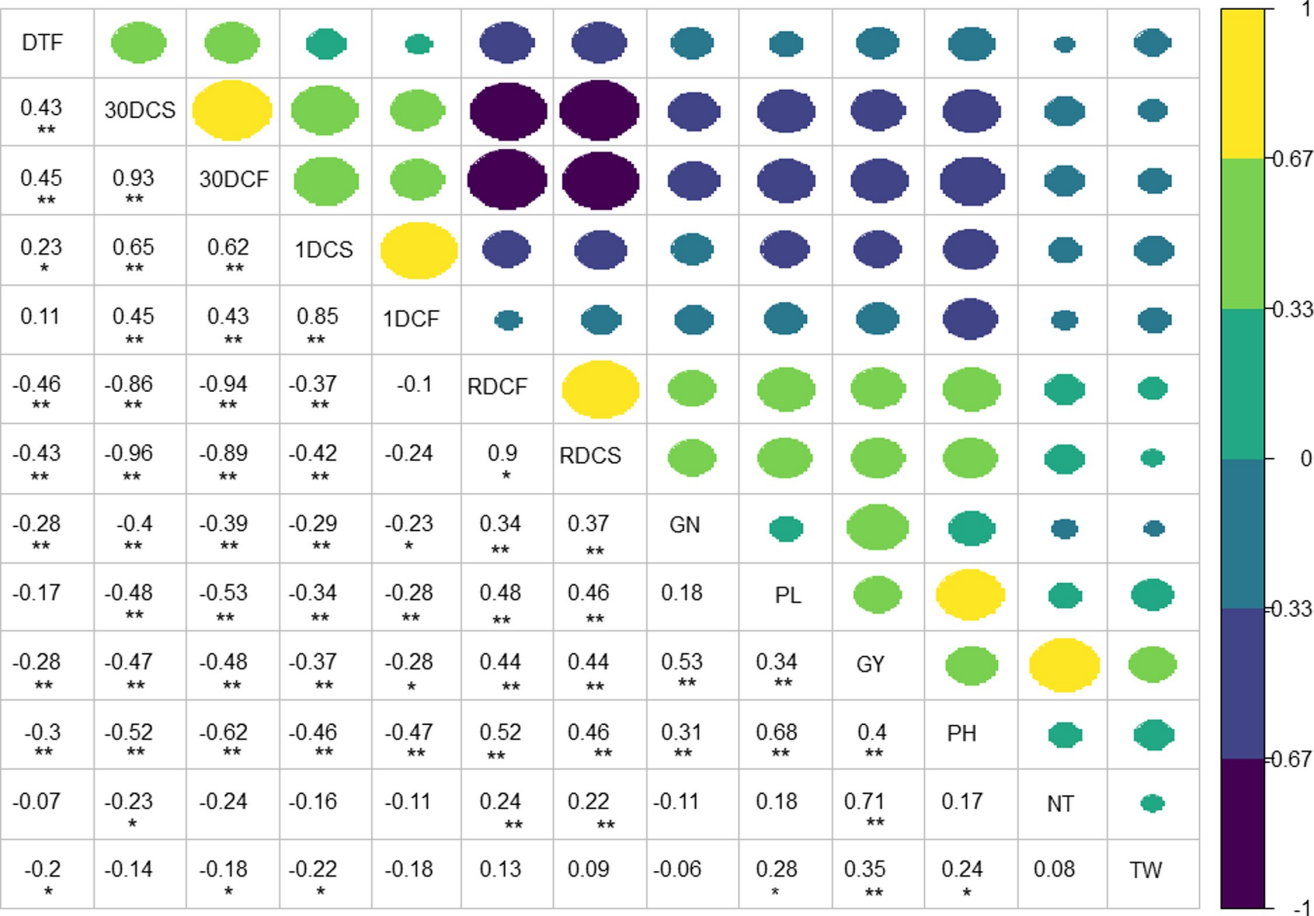
Correlation of delayed leaf senescence with agronomical traits in the BC_2_F_4_ derived population. ** Correlation is significant at the 0.01 level (2-tailed); *Correlation is significant at the 0.05 level (2-tailed).

The important determinant of delayed leaf senescence *i*.*e*., RDCF (reduced decline degree of chlorophyll content of flag leaf) and RDCS (reduced decline degree of chlorophyll content of second leaf) has a significant positive correlation (p<0.05) with grain number per panicle, panicle length, tiller number, plant height and grain yield. However, no correlation was observed with grain test weight and negative correlation was observed with days to heading. Contrastingly, other four components of leaf senescence traits namely, 1DCF, 30DCF, 1DCS and 30DCS showed negative or no correlation with grain number per panicle, panicle length, tiller number, plant height and grain test weight (Fig 1).

### QTL analysis for delayed leaf senescence traits

**Fig 2:**
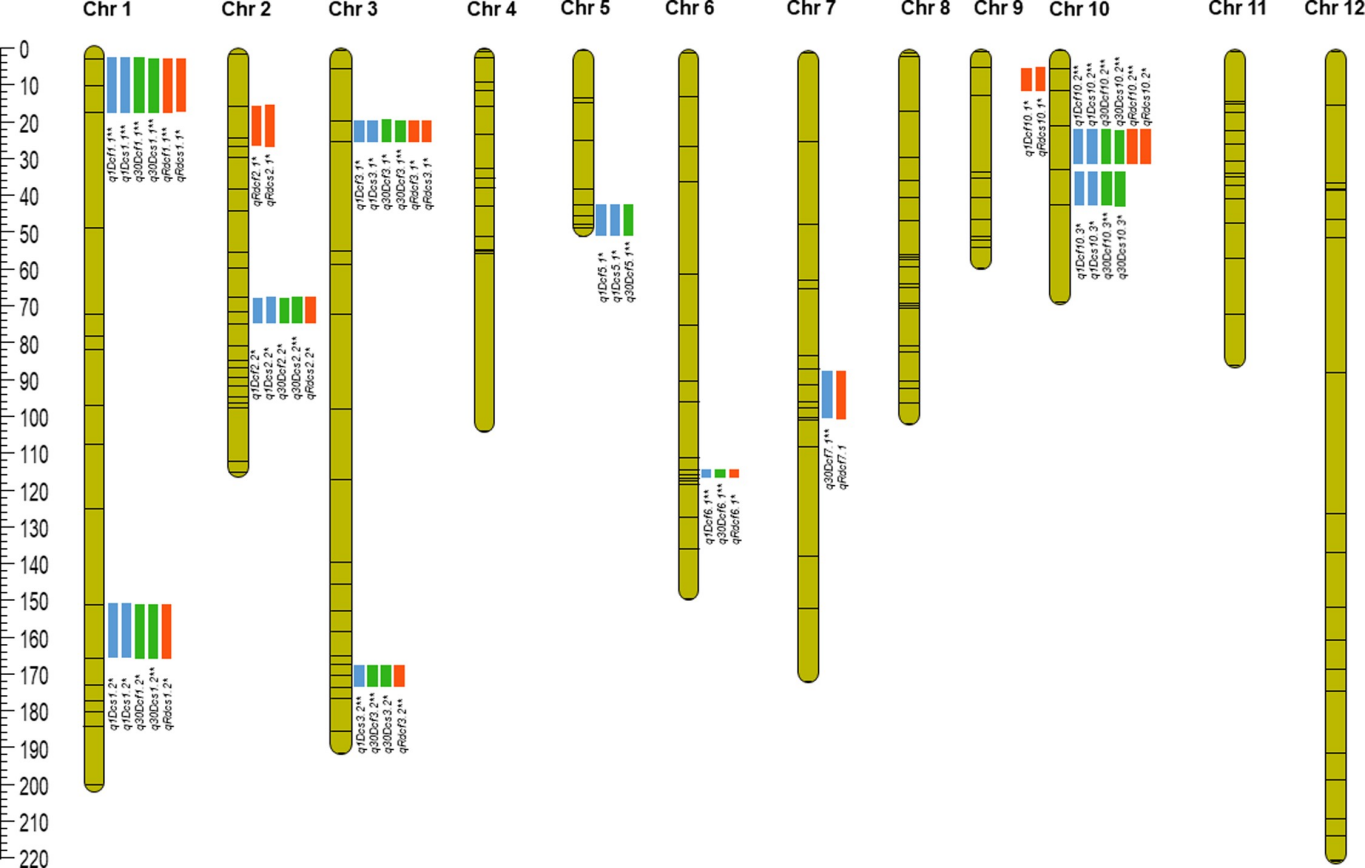
SNP- based genetic map and distribution of QTLs associated with delayed leaf senescence traits. Chromosomes are numbered at the top and QTLs are listed on the right of each chromosome. Various color figures indicate the location of peak LODs of the six traits. QTL detected at heading are marked as * and 30 days after heading are marked as **.

The average polymorphism of 193 SNP markers between BC_2_F_4_ individuals are (AA-74.83%; BB-14.42%; AB-10.75%). QTL analysis by CIM identified a total of 41 QTLs (9 for 1DCF, 10 for 30DCF, 8 for 1DCS, 7 for 30DCS, 5 for RDCF and 2 for RDCS) in WS2016 with LOD value from 2.6 to 7.3 and % PVE (phenotypic variance explained) from 10.3 to 25.1% (Table 2 and Fig 2). Similarly, 29 QTLs (3 for 1DCF, 7 for 30DCF, 3 for 1DCS, 5 for 30 DCS, 6 for RDCF and 5 for RDCS) were identified in DS2017 with LOD value of between 2.5 to 3.7 and % PVE range of 8.2 to 20% (Table 2 and Fig 2). Comparative analysis across the seasons has revealed 21 consistent QTLs (PVE up to 25.1%) for leaf senescence-associated traits and out of them 19 QTLs (2 for 1DCF, 7 for 30DCF, 2 for 1DCS, 5 for 30DCS, and 3 for RDCF) were found as major QTLs (PVE >10%). The identified QTLs for DCF (1DCF and 30DCF) and DCS (1DCS and 30DCS) were contributed by Swarna, while RDCF and RDCS QTLs were contributed by both of the parents (Table 2).

**Table 2:** QTL identification for the six delayed leaf senescence traits in BC_2_F_4_ derived lines.

QTL	Chr^†^	Marker interval			Add^‡^	LOD^€^	R^2^^¥^	Add	LOD	R^2^	Co-localized with previous studies
		left	Peak	right							
						WS2016			DS2017		
1 Degree of chlorophyll content of flag leaf (1DCF)											
*q1Dcf1*.*1*	1	id1000027	id1001073	id1001973	3.6	4.6	0.17	0.4	2.9	0.16	-
*q1Dcf1*.*2*	1	id1021344		id1012929	3.5	4.1	0.15	-	-	-	[12]
*q1Dcf2*.*2*	2	id2006486	id2006643	id2007213	5.5	3.7	0.13	-	-	-	-
*q1Dcf3*.*1*	3	id3000757		id3000946	3.9	4.5	0.16	-	-	-	[9]
*q1Dcf5*.*1*	5	id5007323	id5003638	id5013100	8.1	3.5	0.13	-	-	-	[13]
*q1Dcf6*.*1*	6	id6015035	id6010515	id6011613	7.7	4.7	0.18	1.7	2.7	0.09	[34]
*q1Dcf10*.*1*	10	id10000391		id10001118	5.8	5.5	0.20	-	-	-	-
*q1Dcf10*.*2*	10	id10001636		id10002779	4.9	6.5	0.23	2.6	2.5	0.12	-
*q1Dcf10*.*3*	10	id10003620		id10004477	3.8	4.7	0.15	-	-	-	-
30 Degree of chlorophyll content of flag (30DCF)											
*q30Dcf1*.*1*	1	id1000027	id1001073	id1001973	4.3	6.5	0.23	5.2	2.7	0.11	-
*q30Dcf1*.*2*	1	id1021344		id1012929	4.8	3.8	0.14	-	-	-	[30]
*q30Dcf2*.*2*	2	id2006486	id2006643	id2007213	6.1	3.1	0.11	-	-	-	-
*q30Dcf3*.*1*	3	id3000757		id3000946	5.8	6.7	0.23	-	-	-	-
*q30Dcf3*.*2*	3	id3010173	id3005879	id3012786	8.8	4.2	0.15	5.2	2.9	0.16	-
*q30Dcf5*.*1*	5	id5007323	id5003638	id5013100	11.1	3.9	0.15	4.9	2.7	0.10	[33]
*q30Dcf6*.*1*	6	id6015035	id6010515	id6011613	8.4	4.4	0.16	8.2	3.2	0.18	[34]
*q30Dcf7*.*1*	7	id7002907	id7003271	id7003043	7.4	3.1	0.12	7.1	3.3	0.19	[30]
*q30Dcf10*.*2*	10	id10001636		id10002779	4.9	7.3	0.25	4.3	2.9	0.17	-
*q30Dcf10*.*3*	10	id10003620		id10004477	4.4	3.8	0.14	4.3	2.5	0.11	-
1 Degree of chlorophyll content of second leaf (1DCS)											
*q1Dcs1*.*1*	1	id1000027	id1001073	id1001973	2.2	3.1	0.12	-0.5	2.7	0.15	-
*q1Dcs1*.*2*	1	id1021344		id1012929	2.9	3.0	0.12	-	-	-	[30]
*q1Dcs2*.*2*	2	id2006486	id2006643	id2007213	6.1	3.6	0.13	-	-	-	-
*q1Dcs3*.*1*	3	id3000757		id3000946	4.2	5.6	0.19	-	-	-	[7]
*q1Dcs3*.*2*	3	id3010173	id3005879	id3012786	6.6	3.0	0.10	1.1	2.5	0.08	-
*q1Dcs5*.*1*	5	id5007323	id5003638	id5013100	7.8	2.8	0.11	-	-	-	[6]
*q1Dcs10*.*2*	10	id10001636		id10002779	4.1	4.5	0.17	2.6	2.8	0.11	-
*q1Dcs10*.*3*	10	id10003620		id10004477	3.9	3.5	0.13	-	-	-	-
30 Degree of chlorophyll content of second leaf (30DCS)											
*q30Dcs1*.*1*	1	id1000027	id1001073	id1001973	2.5	4.2	0.16	7.4	3.4	0.18	-
*q30Dcs1*.*2*	1	id1021344		id1012929	4.8	3.1	0.11	5.6	2.5	0.11	[30]
*q30Dcs2*.*2*	2	id2006486	id2006643	id2007213	4.4	3.6	0.12	1.6	2.8	0.16	-
*q30Dcs3*.*1*	3	id3000757		id3000946	4.3	5.2	0.18	4.1	2.4	0.11	-
*q30Dcs3*.*2*	3	id3010173	id3005879	id3012786	6.7	2.8	0.10	-	-	-	-
*q30Dcs10*.*2*	10	id10001636		id10002779	2.9	4.8	0.18	1.1	2.9	0.12	-
*q30Dcs10*.*3*	10	id10003620		id10004477	3.5	3.2	0.11	-	-	-	-
Relative decline in degree of chlorophyll content of flag leaf (RDCF)											
*qRdcf1*.*1*	1	id1000027	id1001073	id1001973	-3.1	3.1	0.12	0.1	2.7	0.12	-
*qRdcf2*.*1*	2		id2002229	id2001301	16.2	3.0	0.12	-	-	-	-
*qRdcf2*.*3*	2	Id2011110		Id2005230	-	-	-	-0.1	2.5	0.14	-
*qRdcf3*.*1*	3	id3000757		id3000946	-4.9	2.6	0.10	-	-	-	-
*qRdcf3*.*2*	3	id3010173	id3005879	id3012786	10.0	3.7	0.13	-0.1	2.7	0.13	-
*qRdcf6*.*1*	6	id6015035	id6010515	id6011613	-	-	-	-0.2	3.3	0.19	[34]
*qRdcf7*.*1*	7	id7002907	id7003271	id7003043	-	-	-	-0.2	3.0	0.17	[30]
*qRdcf10*.*2*	10	id10001636		id10002779	-2.8	3.3	0.13	0.0	2.8	0.16	-
Relative decline in degree of chlorophyll content of second leaf (RDCS)											
*qRdcs1*.*1*	1	id1000027	id1001073	id1001973	-	-	-	-0.2	3.5	0.19	-
*qRdcs1*.*2*	1	id1021344		id1012929	-	-	-	0.15	3.7	0.20	[30]
*qRdcs2*.*1*	2		id2002229	id2001301	15.5	3.7	0.14	-	-	-	-
*qRdcs2*.*2*	2	id2006486	id2006643	id2007213	-	-	-	0.0	2.8	0.16	-
*qRdcs3*.*1*	3	id3000757		id3000946	-	-	-	-0.1	2.69	0.15	-
*qRdcs10*.*1*	10	id10000391		id10001118	3.4	2.9	0.11	-	-	-	-
*qRdcs10*.*2*	10	id10001636		id10002779	-	-	-	0.03	2.5	0.118	-

^†^Chr: chromosome,

^‡^Add: additive effect,

^€^LOD: Logarithm of Odd value,

^¥^R^2^: Phenotypic variance (%)

### QTL analysis for 1DCF and 30DCF

A total of 9 and 3 QTLs for 1DCF were detected in WS2016 and DS2017 respectively (Table 2 and Fig 2). The 9 QTLs detected for 1DCF in WS2016 explained a PVE up to 23% and 3 QTLs detected for 1DCF in DS2017 explained a PVE up to 16%. Similarly, for 30DCF, 10 QTLs were detected in WS2016 with PVE up to 25.1% and 6 QTLs in DS2017 with PVE up to 19%. Comparative analysis of both the seasons results revealed the presence of 2 major QTLs *viz*., *q1Dcf*_*1*.*1*_ and *q1Dcf10*.*2* for 1DCF and 7 major QTLs *viz*., *q30Dcf1*.*1*, *q30Dcf3*.*2*, *q30Dcf5*.*1*, *q30Dcf6*.*1*, *q30Dcf7*.*1*, *q30Dcf10*.*2* and *q30Dcf10*.*3* for 30DCF stably expressed in both seasons (Table 2).

### QTL analysis for 1DCS and 30DCS

A total of 8 and 3 QTLs were detected for 1DCS during WS2016 and DS2017 respectively (Table 2 and Fig 2). The 8 QTLs detected for 1DCS during WS2016 had explained maximum PVE up to 19.5% and 3 QTLs in DS2017 up to PVE of 15%. For 30DCS, 7 and 5 of the detected QTLs explained a maximum PVE of 18% in both the seasons. Further, 2 major QTLs *viz*., *q1Dcs1*.*1* and *q1Dcs10*.*2* for 1DCS and 5 major QTLs *viz*., *q30Dcs1*.*1*, *q30Dcs1*.*2*, *q30Dcs2*.*2*, *q30Dcs3*.*1* and *q30Dcs10*.*2* for 30DCF stably expressed in both seasons (Table 2).

### QTL analysis for RDCF and RDCS

A total of 5 and 6 QTLs for RDCF were detected in WS2016 and DS2017 respectively (Table 2 and Fig 2). The 5 QTLs detected for RDCF during WS2016 had PVE up to 13.4% and 6 QTLs in DS2017 up to PVE of 19%. Among them, 3 major QTLs *viz*., *qRdcf1*.*1*, *qRdcf3*.*2* and *qRdcf10*.*2* stably expressed in both WS2016 and DS2017.

For RDCS, a total of 2 and 5 QTLs were identified in WS2016 and DS2017 respectively. In WS2016, 2 major QTLs (*qRdcs2*.*1*, *qRdcs10*.*1*) detected for RDCS with PVE up to 14% and 5 major QTLs (*qRdcs1*.*1*, *qRdcs1*.*2*, *qRdcs2*.*2*, *qRdcs3*.*1* and *qRdcs10*.*2*) in DS2017 up to PVE of 20%. However, no stable QTLs were detected for RDCF in the current study.

### Identification of putative candidate genes and SNP analysis

The identified 19 major and stable QTLs (2 for 1DCF, 7 for 30DCF, 2 for 1DCS, 5 for 30DCS, and 3 for RDCF) were localized on 10 genomic regions across 7 chromosomes (Chromosome 1, 2, 3, 5, 6, 7 and 10) (Table 2 and S1 Table in S2 Data). Analysis of 10 genomic regions revealed the presence of several genes ranging from 67 (on genomic region 3.1) to 1390 (on genomic region 5.1) (S2 Table in S2 Data) and was grouped into various functional categories using MapMan (S2 Fig in S1 Data). MapMan visualization indicated the presence of diverse categories of genes such as cell cycle and cell division, DNA synthesis, stress responsiveness, regulation of transcription, developmental processes (cell division, flowering etc.), protein modification, protein degradation etc., within the identified QTLs region.

Genes involved in the regulation of chlorophyll metabolism were selected through *in silico* analysis and literature mining. Five potential candidate genes from four genomic regions *viz*., Pectinesterase (*Os01t0743200*) on genomic region 1.2, Oxidoreductase (*Os02t0511100*) on genomic region 2.2, dehydrogenase/reductase (*Os06t0590301*) and Senescence-associated protein 5 (*Os06t0653100*) on genomic region 6.1 and Chlorophyllase (*Os10t0419600*) on genomic region 10.3 associated with the target traits were identified (Table 3). Analysis of SNP in the coding region of identified genes among Swarna and Moroberekan was carried out from available sequence data using RiceVarMap v2.0 browser (http://ricevarmap.ncpgr.cn/v2/). However, no SNPs were detected in selected genes among the parents.

**Table 3:** List of five candidate genes identified in four genomic regions.

S. No	Genomic region	QTL	Gene Locus ID	Start Position (bp)	End Position (bp)	Putative Function (RAP-DB annotation)
1	Genomic region 1.2	*q1Dcf1*.2*	Os01t0743200	31056061	31058475	Similar to Pectinesterase.
		*q1Dcs1*.2*				
		*q30Dcf1*.2*				
		*q30Dcs1*.2**				
		*qRdcs1*.2*				
2	Genomic region 2.2	*q1Dcf2*.2*	Os02t0511100	18277595	18286711	Similar to Oxidoreductase, short chain dehydrogenase/reductase family protein, expressed.
		*q1Dcs2*.2*				
		*q30Dcf2*.2*				
		*q30Dcs2*.2**				
		*qRdcs2*.2*				
3	Genomic region 6.1	*q1Dcf6*.1**	Os06t0590301	23181263	23184271	Similar to short-chain dehydrogenase/reductase (SDR) family protein.
		*q30Dcf6*.1**				
		*qRdcf6*.1*				
			Os06t0653100	26752554	26755222	Senescence-associated protein 5.
4	Genomic region 10.3	*q1Dcf10*.3*	Os10t0419600	14763501	14765095	Chlorophyllase family protein.
		*q1Dcs10*.3*				
		*q30Dcf10*.3**				
		*q30Dcs10*.3*				

* QTL expressed in one season;

** QTL expressed in both season

### Expression profiling of putative candidate genes for delayed leaf senescence

**Fig 3:**
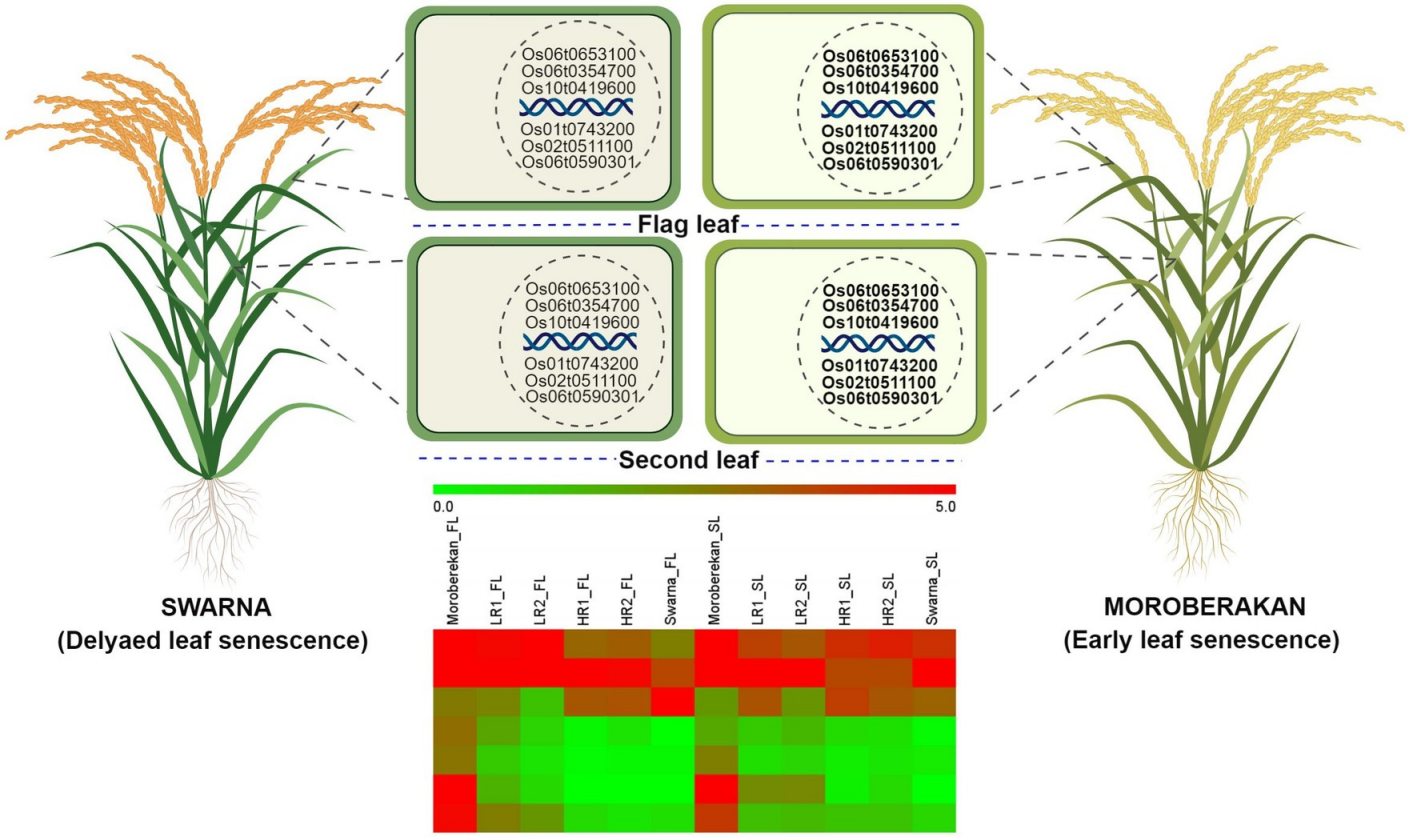
Gene expression model. Expression of five genes (1) *Os01t0743200* (Pectinesterase) (2) *Os02t0511100* (Oxidoreductase, short chain dehydrogenase/reductase) (3) *Os06t0590301* (short-chain dehydrogenase/reductase) (4) *Os06t0653100* (Senescence-associated protein (5) *Os10t0419600* (Chlorophyllase family protein) in flag and second leaf of Swarna (less senescent) and Moroberekan (more senescent) along with two early senescence and two delayed senescence lines. Bold letter represents higher expression and a normal letter represents less expression. Heatmap of six genes in flag and second leaf of Swarna, Moroberekan and phenotypic extreme BC_2_F_4_ derived lines *viz*., ELS-early leaf senescence and DLS- delayed leaf senescence. Expression is shown as log2 (FC) relative to second leaf of early senescence parent. Red marks genes that are upregulated and green for downregulated. The y-axis shows genes that co-regulate based on their directionality and magnitude. The x-axis shows different according to the legend.

Expression profiling of selected five genes associated with leaf senescence was analyzed in FL and SL at 30 days after heading (DAH) of Swarna (the early senescence parent) and Moroberekan (the delayed senescence parent), as well as extreme backcross inbred lines (BILs) having early and delayed senescence nature. The reduction in chlorophyll content of FL and SL of ELS lines IR91648-B-306-B (FL-26.05%, SL-28.67%) and IR91648-B-373-B (FL-24.83%, SL-28.04%) was higher than Moroberekan. The reduction in chlorophyll content of FL and SL of DLS lines IR91648-B-201-B (FL- 8.69%, SL- 17.31%) and IR91648-B-67-B (FL-9.83% and SL- 8.38%) was lower than Swarna. To get insight into the expression variation in the selected five genes, we compared the expression of Swarna and the two early senescence type BILs with Moroberekan and the two delayed senescence type BILs in FL and SL respectively (Fig 3). As expected, all the selected genes showed a significant expression variation between the two classes of senescence. In brief, Pectin Methylesterase 5 (*Os01t0743200-02*) showed 4.1 and 2.5-fold higher expression in Moroberekan than in Swarna in FL and SL respectively. Similarly, oxidoreductase, short-chain dehydrogenase/reductase (*Os02t0511100-00*) and short-chain dehydrogenase/reductase (SDR) (*Os06t0590301-01*) showed a significant higher expression in Moroberekan (6 and 19.3 fold higher in FL and 6 and 19.3 fold higher in SL respectively as compared to Swarna. Similarly, the senescence-associated protein 5 (*Os06t0653100-01*), and a chlorophyll degrading enzyme (*Os10t0419600-00*) gene showed expression differences of 6.3 -fold, and 7.7 -fold in FL respectively, and 19.9 –fold, and 5.0 –fold in SL respectively between Moroberekan and Swarna. As expected, a similar expression pattern was also observed between the early leaf senescence (ELS) type BILs and the delayed leaf senescence type BILs as identified in their respective parental senescence types.

## Discussion

In the current study, it was observed that the SPAD value of FL and SL was higher at heading which gradually diminished subsequently in all plants. This reduction was slower (13.7% and 18.5%) in FL and SL of Swarna as compared to Moroberekan (30.7% and 30.3%). This slow reduction of chlorophyll content in the leaf of Swarna suggests its delayed leaf senescence type. Based on classification given by Thomas and Howarth [10], Swarna can be considered as type B functional stay green which entails a slower decrease in the rate of photosynthesis as compared to Moroberekan. The slower decline in chlorophyll content and more photosynthates translocation in Swarna than functional stay-green cultivar Fedearroz 50 has also been reported [29]. In the current study, a molecular genetics approach involving QTL mapping and expression profiling of the selected potential candidate genes was conducted.

The two important determinants of delayed leaf senescence *i*.*e*., RDCF and RDCS showed significant positive correlation with grains number, panicle length, tiller number, plant height and grain yield except days to heading and test weight. The negative correlation of days to heading with RDCF/RDCS might be due to contribution of alleles from late maturing parent Swarna which is also a contributor of stay green trait. However, negative or no significant correlation of DCF and DCS with yield traits was observed. The positive correlation of stay green and yield-related traits in rice was only reported in intra-subspecies population SNU-SG1/Ilpumbyeo (*japonica-japonica*) [4] and Akenohoshi / Koshihikari (*japonica-japonica*) mapping population [5] in earlier conducted studies. In other studies, negative correlation of stay green traits with grain yield was reported in inter-subspecies crosses (*japonica*–*indica*) [11,30,31]. This negative correlation in *indica-japonica* crosses might be due to the presence of wide compatibility genes in *japonica* donors and their segregation in the mapping population leads to panicle sterility.

### Effect of inter-subspecies cross on fertility/sterility

Despite several reports on negative correlation of stay green traits with yield characteristics in inter-subspecific populations in rice, in the present study we identified positive association with yield attributing parameters (GNP, PL, NT and GY). The negative correlation of stay green with yield attributing parameter in inter-subspecies crosses may be due to partial sterility occurring in a portion of lines due to the presence or absence of wide compatibility (*WC*) gene(s). Partial sterility results in hindrance of nutrient (carbohydrate, nitrogen) transport and movement from leaf to seed part which might result in lower seed setting and greener leaf due to higher chlorophyll content as a result of higher nitrogen content. To avoid sterility problems in the present study, as Moroberekan possesses wide compatibility (*WC*) gene, we discarded lines which showed sterility more than 35% as the population under study is also derived from inter-subspecies crosses. Sterility more than 35% are considered as sterile [32] and are not considered in the study. In the previous studies, this sterility caused due to the segregation of WC gene might have not been considered or they were overlooked while determining the correlation among yield and stay green traits.

### QTL analysis for delayed leaf senescence trait

For delayed leaf senescence traits, a total of 41 QTL regions (PVE up to 25.1%) and 29 QTL regions (PVE up to 20%) were identified in WS2016 and DS2017 respectively (Table 2). Interestingly, all the stable major-effect QTLs identified for delayed leaf senescence traits were co-localized in 10 QTL regions (S1 Table in S2 Data). Identified QTLs on genomic region 1.2 of chromosome 1 (24.40–36.16) were co-localized with previously identified QTLs namely, *QICb1a* (27.33–25.45) and *QICb1b* (20.06–27.19) associated with leaf chlorophyll b at heading [30] and *QRdg1* (32.98–37.88) [12]. The QTL identified on genomic region 3.1 of chromosome 3 (1.31–1.71) has previously reported QTLs, *QICb3* for leaf chlorophyll b at heading [9] and QTL, *NTR3* for nitrogen transport rate 21 days after heading [5]. The QTLs identified on genomic region 5 of chromosome 5 (18.32–27.00) were co-localized with previously identified QTLs for degree of chlorophyll content of flag leaf *viz*., *1DCf5*, *1DCs5*, *1DCfs5*, *50DCf5* and *50DCs5* (23.97–27.31) [33]. Identified QTLs on genomic region 5.1 of chromosome 5 (18.32–27.00) were co-localized with previously identified QTLs, *dcf5* for degree of chlorophyll content of flag leaf and *dcfs5* (19.91–18.69) for degree of mean chlorophyll content of the flag and second leaves [4]. QTLs identified on chromosome 6 (27.68–22.32) has already reported QTLs, *qLDLJ-6-3* for number of late-discoloring leaves per plant at 25 DAF [34]. Similarly, for QTLs reported on genomic region 7.1 of chromosome 7 (18.62–20.17) were co-localized with previously identified QTLs namely, *QIICa7b* (16.73–19.25) for leaf chlorophyll content at maturity; *QII/ICb7a* and *QII/ICb7a* (16.73–19.25) (Table 2 and S3 Table in S2 Data). Co-localization of QTLs for delayed leaf senescence traits, could be due to linkage and/or (more likely) pleiotropic. From a physiological point, co-localization of QTLs can be understood as the component of delayed leaf senescence traits are also functionally related with each other and eventually regulate the greenness of leaves *viz*., DCF, DCS, RDCF and RDCS.

One yield QTLs, *qDTY*_*1*.*1*_ identified previously in same population [24], on chromosomes 1 were positioned at the same genomic locations of *q1DCF*_*1*.*2*_ QTL for delayed leaf senescence traits. The identified QTL regions for DLS can be promising targets for improvement of rice yield through marker-assisted introgression of the delayed leaf senescence trait. The increased yield under drought by *qDTY*_*1*.*1*_ might be due to delayed leaf senescence under drought not studied till now. Similar to this finding, two-grain yield QTLs (*Yld6* and *Yld9*) were previously reported in the same positions of the stay green QTLs *Csfl6* and *Tcs9* in two RILs populations obtained from the combination of “Suweon490” (*japonica* and synchronized) x “SNU-SG1” (japonica and SG) and “Andabyeo” (*indica* and synchronized) x “SNU-SG1” [13].

### Candidate gene analysis

Expression analysis of selected 5 genes identified from 10 stable and major effect QTL regions showed differential transcript level in the FL and SL of Moroberekan, Swarna and selected ELS, DLS BIL lines. The higher expression of Pectin methylesterases 5 (*OsPME5*) (*Os01t0743200*) in FL and SL of Moroberekan as compared to Swarna might play a role in inducing early senescence. The strong expression of *OsPME1* was reported during senescence of detached rice leaves [35]. Overexpression of *OsPME1* resulted in higher levels of jasmonic acid and its derivatives jasmonate, which in-turn reported as senescence promoting substance [36].

The higher of Oxidoreductase, short-chain dehydrogenase /reductase (*OsCDR*) (*Os02t0511100*) and short-chain dehydrogenase /reductase (*Os06t0590301*) in FL and SL of Moroberekan might play role in promoting early senescence. *OsCDR* represents a Chl b reductase, necessary for catalyzing the first step of Chl b degradation and thereby promoting early senescence [37,38].

The higher expression of Senescence-associated protein 5 (*Os06t0653100-01*) in FL and SL of Moroberekan link with delayed leaf senescence. Genes up-regulated during senescence, designated as senescence-associated genes (SAGs) have been identified in various species [5,39–45]. They are involved in macromolecule degradation, nutrient recycling, defense and cell rescue mechanisms, transcriptional regulation and signal transduction [43].

The expression of Chlorophyllase (*Os10t0419600-00*) gene was higher in FL and SL of Moroberekan. Increase in chlorophyllase activity has been reported during the senescence of excised leaves [46]. Ben-Yaakov et al. [47] compared the changes in chlorophyllase (Chlase) activity and chlorophyll content during leaf senescence in eleven plant species. Contrary to expectation, Chlase activity does not increase during leaf senescence but rather declines in most plant species examined. Chlorophyllase (Chlase) hydrolyzes Chl a, forming chlorophyllide a (Chlide a) and phytol, and is thought to catalyze the first step of Chl a degradation [48,49].

In recent genome wide association analysis (GWAS) study 25 known genes, among which the *OsSG1* accounted for natural variation in chlorophyll content and stay green. Significant phenotypic differences between alleles are caused by non-synonymous SNPs within six known genes and three SNPs in the promoter of *OsSG1* were also reported [50]. In current study, lack of structural variation/SNPs in the coding region of five genes sequence between both the parents were reported. We speculated that the variation in delayed senescence is not due to structural variation in the studied genes and might be due to methylation or copy number, or other variation change which leads to change in the expression of the targeted candidate genes. However, the involvement of other genes (not functionally characterized) might not be ruled out.

### Implication of identified QTLs and superior lines in breeding

The important genomic region 6.1 on chromosome 6 which possess stable QTLs for 1DCF, 30DCF and RDCF, possess two genes oxidoreductase, short-chain dehydrogenase /reductase (*Os06t0590301*) and senescence-associated protein 5 (*Os06t0653100*) which can be ideal candidate genomic region for utilization in breeding. The other candidate genomic region (1.1 and 10.2) identified on chromosome 1 and 10 respectively, harbor stable effect QTLs for almost all studied traits except RDCS (observed in only one season) can also be useful. However, the five genes studied for validation are not located in the above two genomic regions. This entails that there might be some other regulatory genes on genomic region 1.1 and 10.2 playing roles in delaying leaf yellowness that were not studied previously. Six lines (IR91648-B-191-B, IR91648-B-229-B, IR91648-B-374-B, IR91648-B-91-B, IR91648-B-235-B, IR91648-B-257-B) with these three genomic regions coupled with reduced RDCF, RDCS and good yield were identified (S4 Table in S2 Data). All these QTLs and identified lines possessing favorable alleles can be deployed after validation for improving above mentioned traits through molecular breeding.

## Conclusion

Ten stable QTL regions were identified for six delayed senescence traits. Co-localization of QTLs for different delayed senescence traits indicate the presence of a partly common genetic basis which might be due to tightly linked loci or the pleiotropic effects of a single and/or a set of QTLs controlling these traits. One yield QTL, *qDTY*_*1*.*1*_ on chromosomes 1 positioned at the same locations with the *q1DCF*_*1*.*2*_ QTL for DLS trait strengthens the association between the presence of DLS and high productivity in rice. Expression analysis of identified genes further supports their role in controlling leaf senescence in rice. Significant positive correlation of two most important determinants of delayed leaf senescence traits namely, RDCF and RDCS with grain number per panicle, panicle length, tiller number, plant height and grain yield indicate their usefulness in increasing yield by increasing source-sink strength. Further efforts are required in the coming years to dissect the complex regulatory networks associated with delayed leaf senescence and its impact on source to sink balance.

## Supporting information

S1 Data (PPTX)

S2 Data.w (XLSX)
