# Expression of Concern: Unraveling candidate genomic regions responsible for delayed leaf senescence in rice

**DOI:** 10.1371/journal.pone.0350423

**Published:** 2026-06-01

**Authors:** The PLOS One Editors

The *PLOS One* Editors issue this Expression of Concern because this article [1] is one of a series of articles for which there are concerns about competing interests and the reliability of peer review. The competing interests were not disclosed as required by PLOS policy.

PLOS regrets that the peer review concerns were not identified and addressed before the article was published.

The Editors have no evidence of any author involvement in the peer review concerns for this article.

In following up on this matter, PLOS did not evaluate the article’s integrity or scientific validity.
