# Supplementary material for: Unraveling candidate genomic regions responsible for delayed leaf senescence in rice
Source: PLoS One. 2020 Oct 15;15(10):e0240591. doi: 10.1371/journal.pone.0240591 (PMC7561107; doi:10.1371/journal.pone.0240591)
Supplement: S1 Data — (PPTX) [file pone.0240591.s001.pptx]

## Slide 1
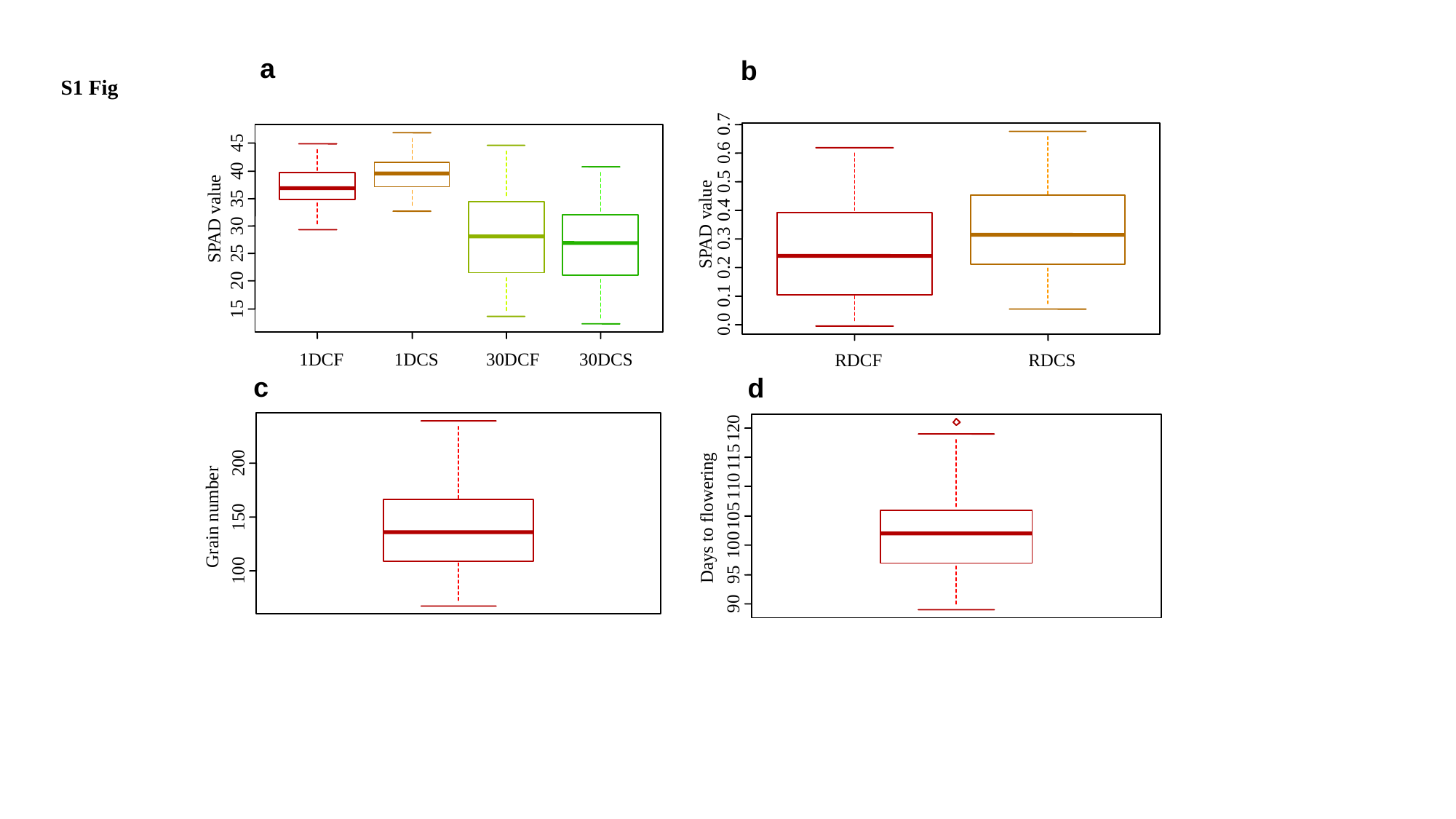

a
b
0.7
0.6
0.5
0.4
0.3
0.2
0.1
0.0
RDCF
RDCS
S1 Fig
45
40
35
30
25
20
15
1DCF
1DCS
30DCF
30DCS
SPAD value
SPAD value
200
Grain number
150
100
120
115
110
105
Days to flowering
100
95
90
c
d

## Slide 2
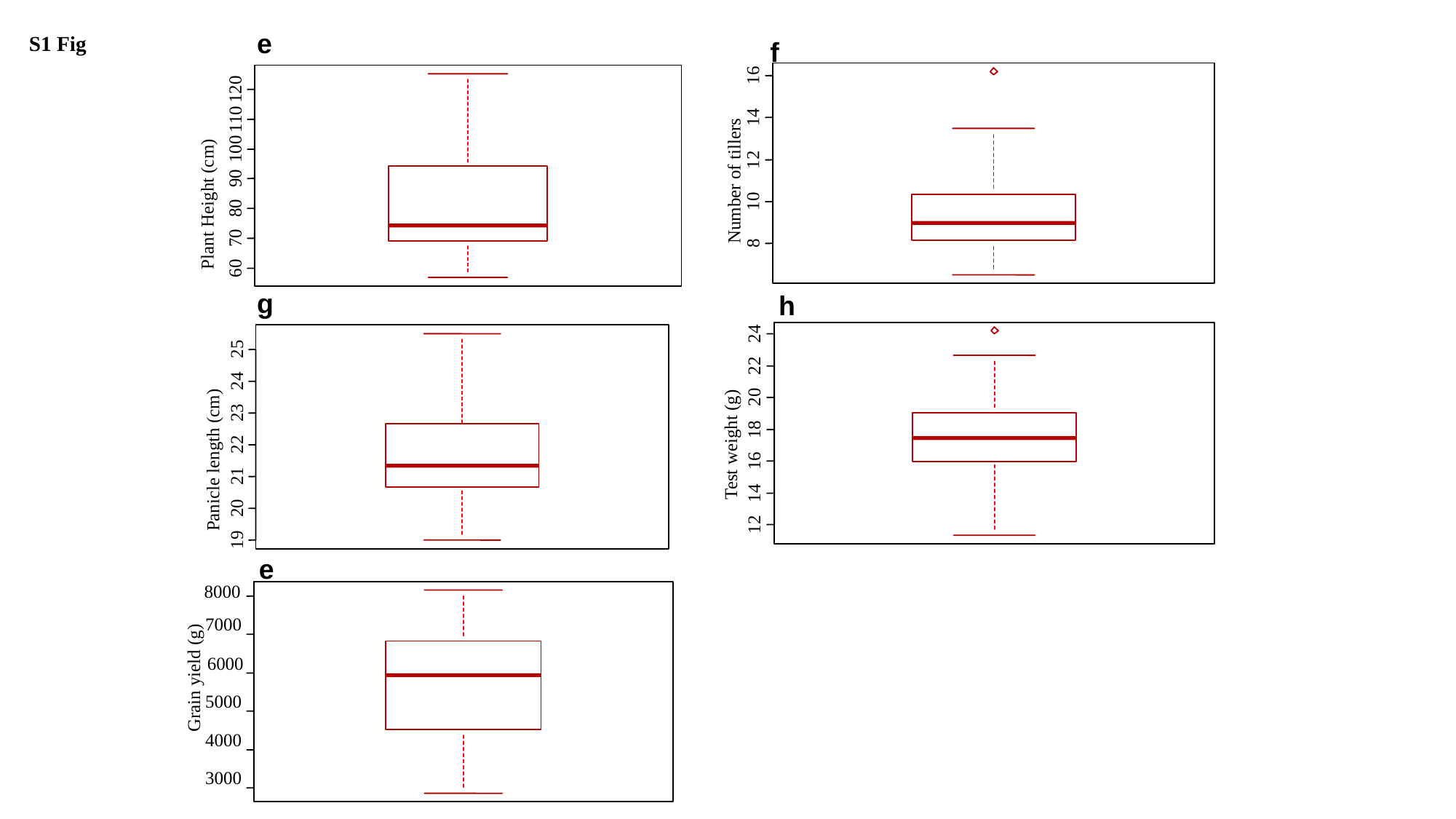

16
14
12
Number of tillers
10
8
e
S1 Fig
f
120
110
100
90
Plant Height (cm)
80
70
60
24
22
20
Test weight (g)
18
16
14
12
25
24
23
22
Panicle length (cm)
21
20
19
g
h
8000
7000
6000
Grain yield (g)
5000
4000
3000
e

## Slide 3
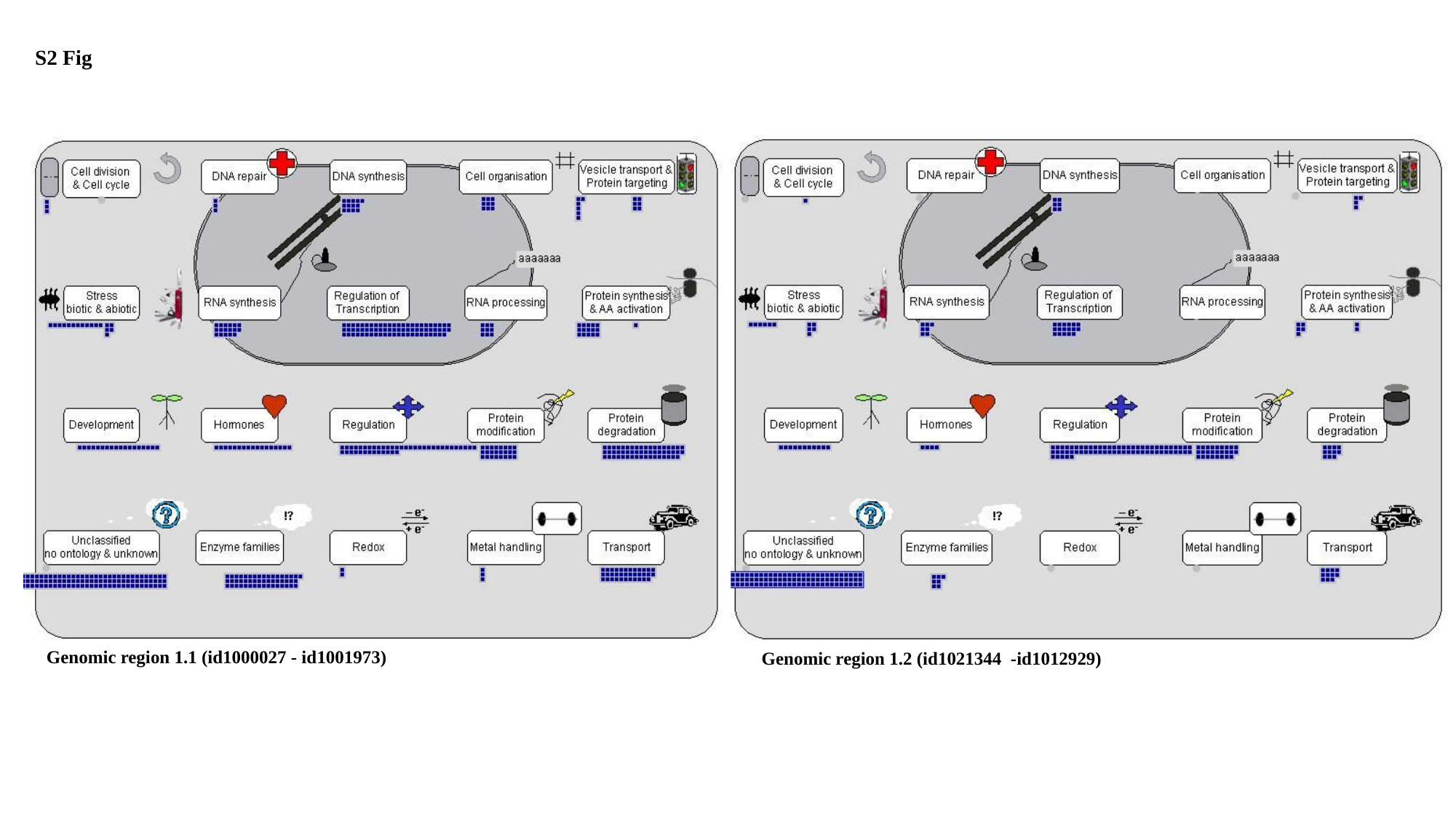

S2 Fig
Genomic region 1.1 (id1000027 - id1001973)
Genomic region 1.2 (id1021344 -id1012929)

## Slide 4
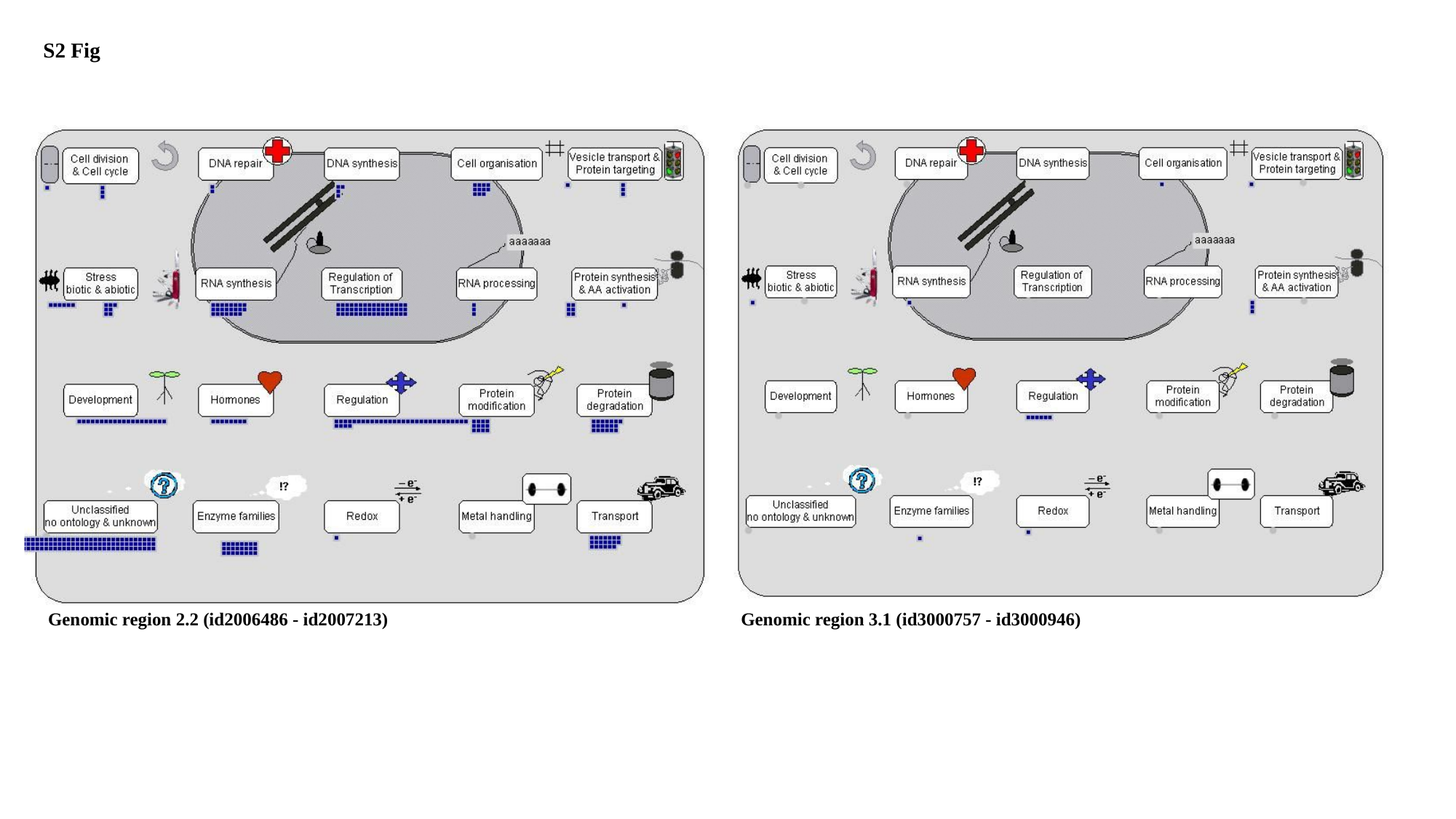

S2 Fig
Genomic region 2.2 (id2006486 - id2007213)
Genomic region 3.1 (id3000757 - id3000946)

## Slide 5
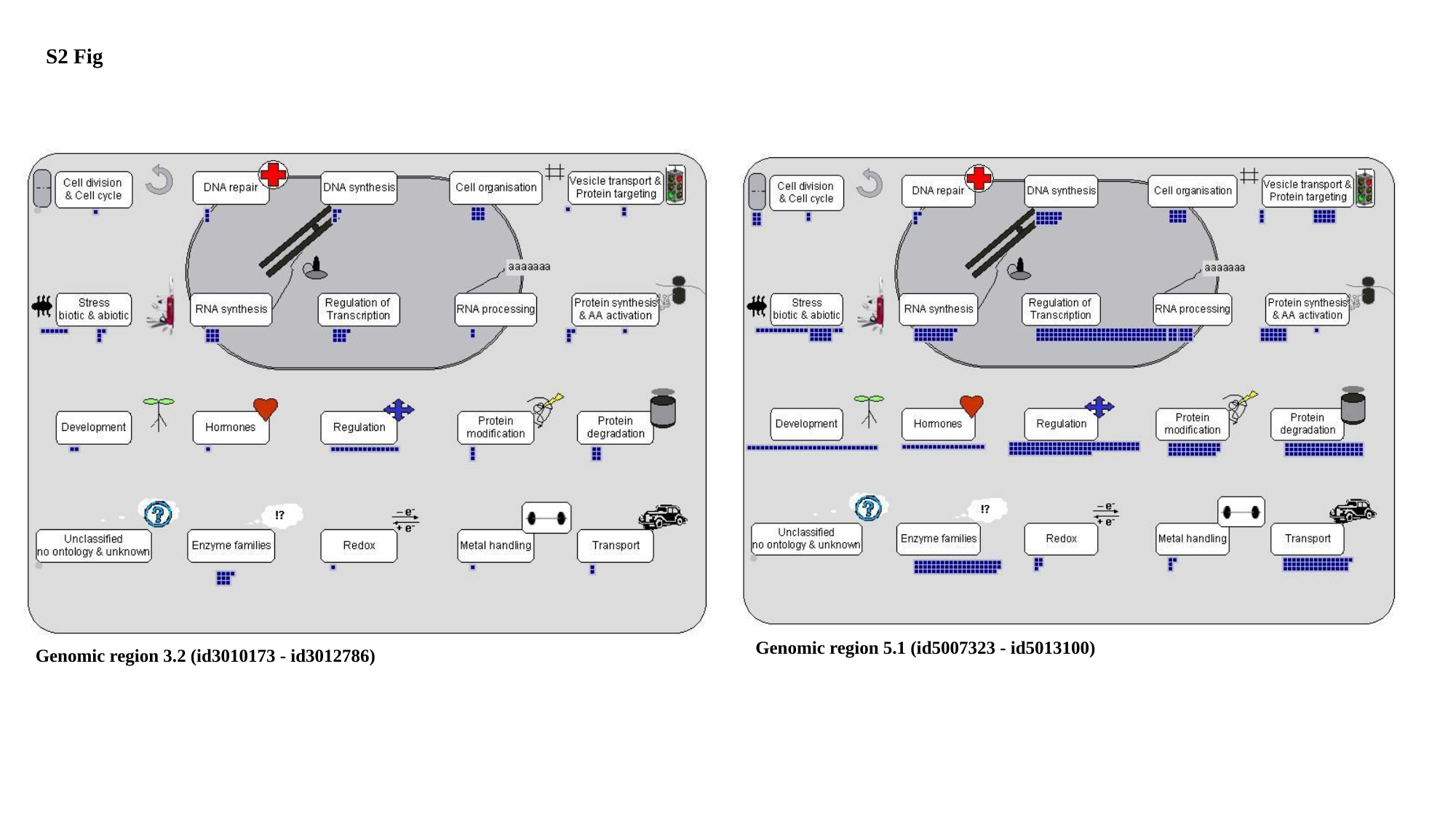

S2 Fig
Genomic region 5.1 (id5007323 - id5013100)
Genomic region 3.2 (id3010173 - id3012786)

## Slide 6
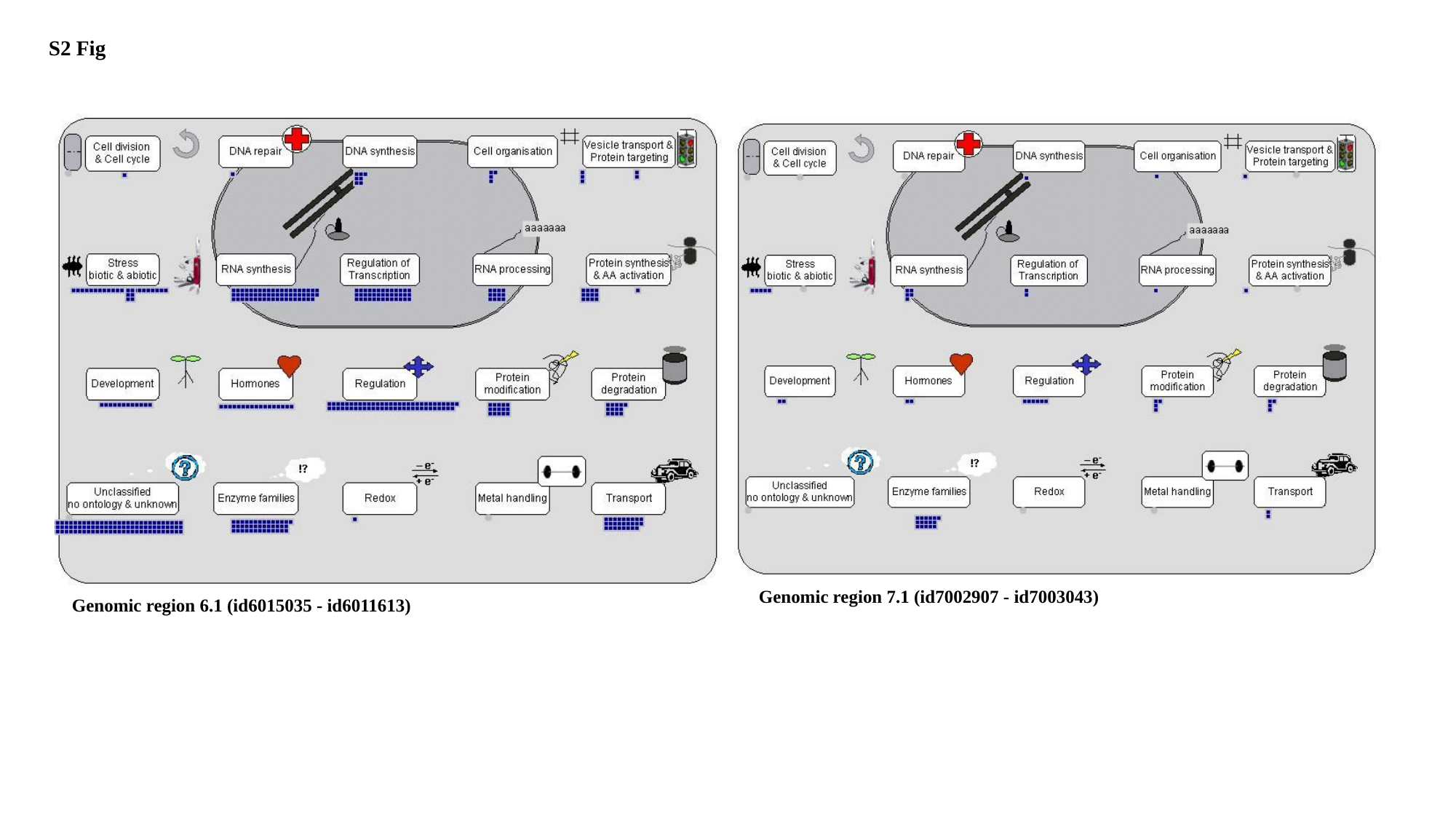

S2 Fig
Genomic region 7.1 (id7002907 - id7003043)
Genomic region 6.1 (id6015035 - id6011613)

## Slide 7
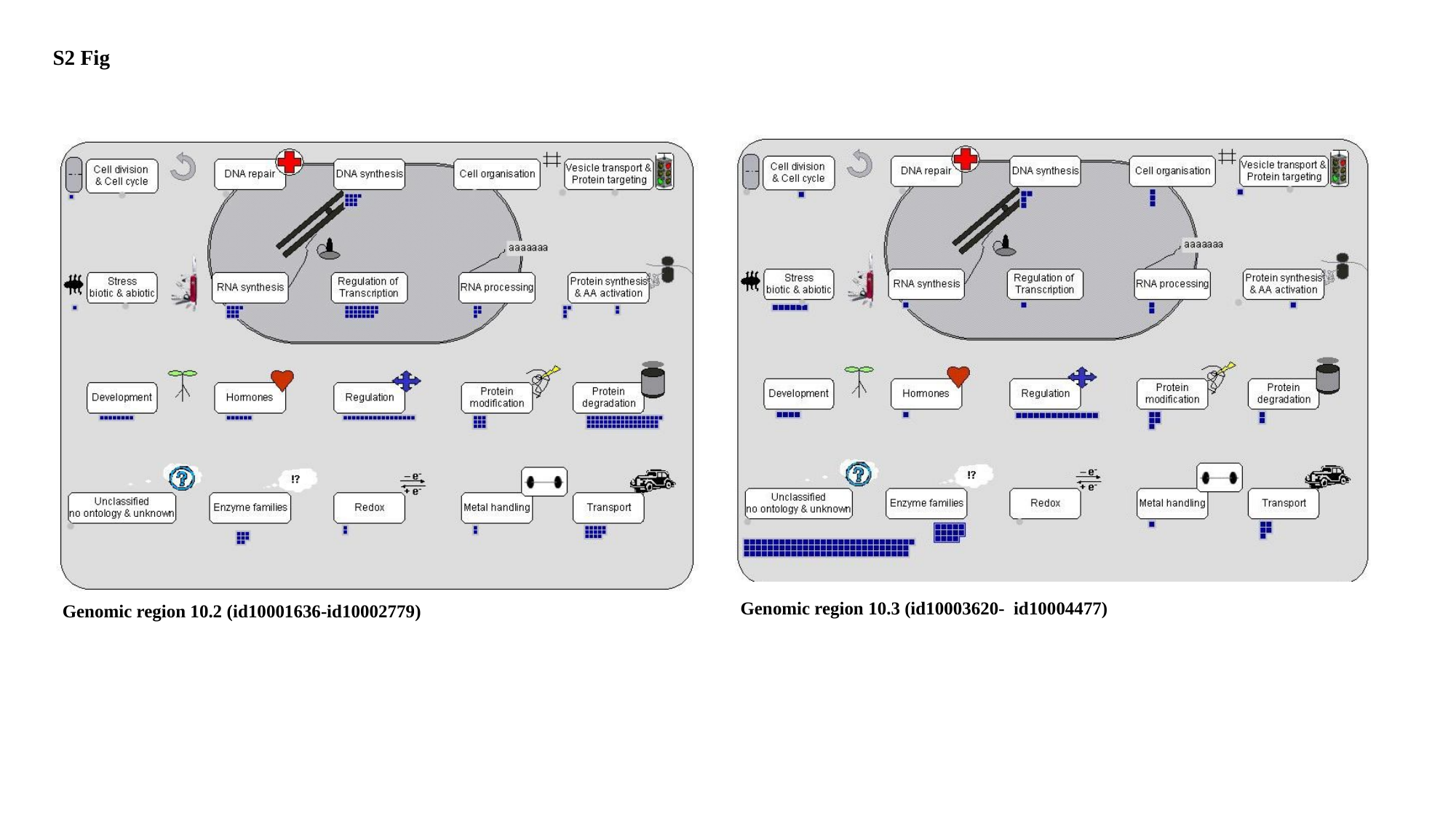

S2 Fig
Genomic region 10.3 (id10003620- id10004477)
Genomic region 10.2 (id10001636-id10002779)
